# Community- based Active Tuberculosis Case Finding in Pastoralist Communities of North-Eastern Uganda

**DOI:** 10.9734/mrji/2019/v29i330166

**Published:** 2019-11-04

**Authors:** Guma Isaac, Emuron John Robert, Namugambe Swabrah, Nabirye Gloria, Philip Denis Okungura, Paul Oboth, Jacob S. Iramiot, Rebecca Nekaka

**Affiliations:** 1 Department of community and public Health, Faculty of Health Sciences Busitema University, Uganda.; 2 Moroto Regional Referral Hospital, Moroto District, Uganda.; 3 Department of Microbiology and Immunology, Faculty of Health Sciences Busitema University, Uganda.

**Keywords:** Tuberculosis, gene xpert, case finding and multidrug resistance

## Abstract

**Background:**

Given the global urgency to improve tuberculosis (TB) case detection, a renewed interest in active case finding (ACF) has risen. Missed TB cases pose a serious threat as they continue to fuel TB transmission in the community. We aimed to assess the feasibility of community based ACF for TB among people living in a pastoralist community in Uganda and determine its impact on case detection and treatment uptake.

**Methods:**

Between April and May 2019, four third year medical and nursing students placed at Moroto Regional Referral for community orientation worked together with community health workers to conduct a door-to-door survey for TB in pastoralist communities of Nadunget Sub County, Moroto district. The community health workers and the Medical/Nursing students performed symptom screening, collected sputum and facilitated specimen transport to the laboratory. Gene Xpert MTB/RIF assay was performed at the regional referral Hospital for all sputum samples. The community health workers were tasked to follow up on all those clients whose samples turned out to be positive so that they could start treatment as soon as possible. All presumptive cases with negative sputum results were referred to the TB clinic for further evaluation.

**Results:**

In one month, we screened 385 individuals and identified 143 aged above 15 years with symptoms suggestive of TB. Among the presumptive cases, 132 (92%) reported a cough of more than two weeks and we were able to obtain sputum samples from 84(58.7%) participants. We diagnosed 11, including 8 bacteriologically confirmed TB cases using Gene Xpert and there was no multidrug resistant case identified. The median time from sputum collection to notification of the positive result was 3 days. All the positive cases were followed up and initiated on treatment.

**Conclusion:**

The findings from our study suggest that in a pastoralist community, ACF for TB using a sensitive symptom screen followed by Gene Xpert contributed to improved case detection of TB, shortening the turnaround time hence timely initiation of patients on TB treatment.

## 1. INTRODUCTION

Tuberculosis (TB) remains one of the most dreaded diseases and leading cause of death from a single infectious agent [1]. It is caused by *Mycobacterium tuberculosis* and its fatality are very distinct in the pages of history. TB affects different ages and is a major contributor to disease burden in most of the developing countries[2]. Despite the progress made in the last decade, Uganda remains one of the countries with the highest TB burden [1]. Missed TB cases pose a serious threat as they continue to fuel TB transmission in the community. TB case notification, which heavily relies on symptomatic individuals voluntarily seeking care at health facilities as advocated for by WHO have stagnated[2]. Several TB prevalence surveys have indicated up to 40% of new TB cases remain undiagnosed despite the implementation of directly observed treatment (DOTS) strategy [3].This Passive case finding strategy has proven to be inadequate to control TB [2].

A number of studies from different settings identified hindrances to early detection of TB which included, geographical or socio-psychological barriers [4], the time taken to access TB care services, the expenses incurred, inadequate funding of health services [5], limited staff capacity, and poor capacity building [6], among others.

The TB burden is high in poor and marginalized communities who face many barriers to access health care services e.g. lack of TB awareness, competing priorities in terms of time and money, long distances to health facilities, and shortage of experienced personnel among others [7]. Karamoja region is a pastoralist community where people live in grass thatched houses (locally known as “Manyattas”) which are poorly ventilated and congested. The long distances moved by these nomadic pastoralists in search of pasture and water, disconnects them from accessing TB services from facilities.

There is need for innovative strategies to complement facility based passive case finding. One such strategy, is active case finding (ACF) which involves systematically identifying individuals in the community with signs and symptoms of active TB. ACF has gained interest in the last decade [7] and aims at reducing barriers to early TB case detection, including delay in presentation to a health facility[8]. ACF reduces the risk of poor treatment outcomes, prevalence of TB related deaths, and transmission of TB by shortening the duration of the infectious period [1].

The revised National TB/Leprosy control program aims to eliminate TB by 2025. To achieve this, early initiation of treatment which, in turn, depends on diagnosis would play a big role. For complete diagnosis of the estimated cases of TB, there is need to identify nearly 40% of missed cases. Active case finding was proposed under the National TB and Leprosy Program (NTLP) to enhance TB case finding[9]. In Moroto, 60% of the total expected cases of TB are notified [10]. An active case finding program has been initiated and prioritized especially for household contacts of active TB cases and people living with HIV.

Despite of the recent recognition of the role of active case finding, there is a dearth of literature about community based active case finding and how best to integrate it to identify undiagnosed TB cases in the community. Therefore, this study aimed to identify the cases of TB through active case finding and to assess feasibility among people living in a pastoralist community of North Eastern Uganda.

## 2. MATERIALS AND METHODS

### 2.1 Study Design and Setting

A community - based, cross sectional, descriptive survey was conducted in three parishes of Nadunget sub county, Moroto District. Moroto is situated in the north eastern region of Uganda and covers a total area of 3537.6sq km with a total population of 103,432 people. Nadunget is made up of six parishes i.e. Acherer, Komaret, Loputuk, Lotirir, Nadunget and Naitakwae. Nadunget has approximately 42,662 people. The area is highly inhabited by the Karimojong who are the main inhabitants of the rangelands. The Karimojong are mainly nomadic pastoralists and a distinctive ethnic group that highly cherishes its traditions and norms. This area is well connected to primary health care facilities including Nadunget Health center III at the sub county and two health center IIs (Lotirir and Acherer). The area also has a tertiary care government facility (Moroto Regional Referral Hospital) (10 km) where adequate diagnostic and laboratory services related to TB are made available free of charge.

### 2.2 Study Population and Sample Size Determination

Between April and May 2019, 385 adults were enrolled and consented to participate from 13 villages from the 3 most populated parishes of Nadunget subcounty.

The study community comprised of individuals aged 15 years and above living mainly in crowded “Manyatas” and highly populated with very poor ventilated houses. The study was conducted on a household basis involving all residents of the household present at the time of data collection.

### 2.3 Inclusion and Exclusion Criterial

All consenting adults aged 15 or more were interviewed using a standardized questionnaire in the local language with the help of an interpreter. The cases detected during the survey who were already on TB treatment were not considered as actively detected cases.

### 2.4 Screening for TB

Trained medical undergraduate students, assisted by community health workers performed household-to-household visits to screen available household members for TB symptoms as defined in the intensified case finding (ICF) form as any cough, unintentional weight loss, fever, night sweats or hemoptysis. If a client reported any symptom, they were classified as presumptive case and were taken through clinical assessment. All consenting adults aged 15 or more with a positive TB symptom screen were interviewed using a standardized questionnaire in the local language. The cases detected during the survey who were already on TB treatment were not considered as actively detected cases. This study was conducted as a part of the community-based education research and service program for the medical undergraduates.

### 2.5 Clinical Algorithm

In our ACF programme, all samples were tested by GeneXpert. For logistical and financial reasons, we did not screen using the chest radiography. If patients were unable to provide sputum, they were referred to the Hospital for further assessment as per national policy.

### 2.6 Laboratory Procedure

#### Sputum collection:

The revised national TB program currently recommends all presumptive individuals to be evaluated with sputum specimens. The Medical students together with the community health workers collected the specimens at home, picking one sample on the spot. Patients were given instructions on how to produce a good quality sputum specimen following a standard operating procedure. Specimens were transported daily to Moroto Regional Referral hospital, where all cases were registered in the TB presumptive Register. Fresh specimens for Gene Xpert were packed in secured, cool boxes and brought to Moroto regional referral Hospital.

#### Lab test:

Gene Xpert testing was done as per standard operating procedures. A single Gene once. For rifampicin resistant cases on Gene Xpert testing, a MTBDRPlus assay was requested to rapidly confirm isoniazid and rifampicin resistance at the laboratory of Moroto Regional Referral Hospital. All clients were offered free HIV counselling and testing.

### 2.7 Data Management and Analysis

The information collected was compiled processed, checked thoroughly for any errors made. The data was entered in STATA version 14.0 to check for any errors and then summarized using measures of central tendencies like mean, median and then presented using tables, pie charts and relevant graphs. Data collected from focus group discussions and open-ended questions was validated and grouped accordingly. The descriptive demographic statistics were presented in tables or pie charts.

## 3. RESULTS

### 3.1 Socio-demographic Characteristics of the Study Population

A total of 385 individuals aged ≥15 years were screened for pulmonary Tuberculosis through Xpert assay was done on a random spot specimen. Inconclusive results were repeated symptomatic screening using an intensified case finding tool incorporated into an interviewer administered questionnaire. 145 males (37.7%) and 240(62.3%) females participated took part in the study with male to female sex ratio of 1:1.7. The Average age of the study population was 40 years with a standard deviation of ±21 years, ranging from 15 to 90 years. About 264 (68.6%) had never attended any formal education whereas 86 respondents (22.3%) reported to have stopped in primary level. Only 7 (1.8%) graduated from a higher institution of learning.

Of the total respondents, 289 (75.1%) live in Manyattas which are poorly ventilated 88(22.9%) respondents live in average semi-permanent houses, whereas only eight (2.0%) lived in permanent houses with proper ventilation status, 258 (67.0%) individuals were unemployed, whereas only 2.3% were salary earners.

#### Prevalence of undiagnosed TB:

Of the 385 respondents who took part in the study, 143 (37%) cases were found to have at least one symptom suggestive of TB. Among the presumptive cases, 132 (92%) reported a chronic cough for more than 2 weeks. Mean age of the presumptive cases was significantly more compared to the general population. Of the 143 presumptive cases, 84 (58.7%) individuals provided a sputum sample on spot and all samples were tested using the GeneXpert. Those who could not produce sputum were referred to the health center for evaluation by the clinician. Of the 84 sputum samples tested eight had, MTB detected. Those with negative GeneXpert were referred to the hospital for further evaluation and three were clinically diagnosed with TB. Therefore, the prevalence of newly diagnosed Pulmonary TB was 2857 per 100,000 persons in individuals aged ≥15 years.


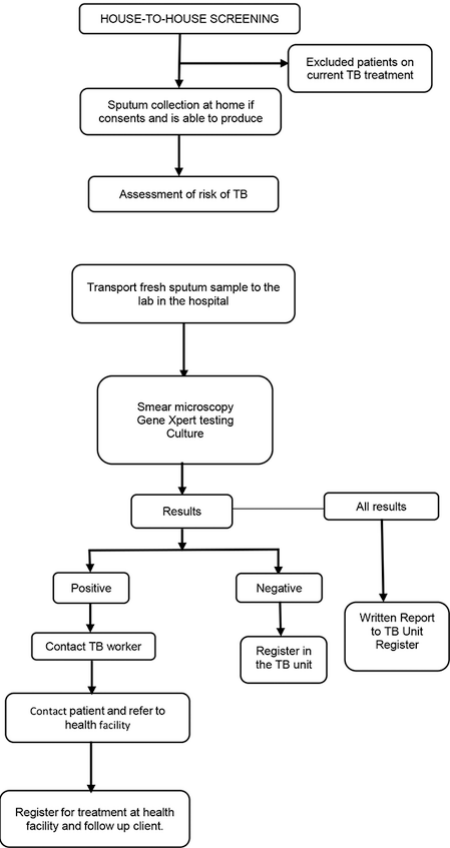


Schematic representation of the community based active TB case finding approach

Of the 33 (23.1%) individuals were referred to Moroto regional referral hospital for further clinical investigations, 6 turned up for assessment and 3 were clinically diagnosed for Extra pulmonary TB and initiated on treatment. A total of 23 (16%) of the presumptive individuals had a chronic cough≥2 weeks, but could not provide a sputum sample at the time of data collection due to lack of productive cough during mid-day time and 3(2.1%) declined to produce sputum deliberately even after consent to be screened. There were more elderly TB positive clients in the age group > 60 years, than any other age group.

## 4. DISCUSSION

For a period of one month, we performed a household-to-household active TB screening in pastoralist communities of Nadunget sub county, Moroto District reaching a population of 385. We identified 143(37%) presumptive cases of TB and 84 (58.7%) were able to produce sputum for analysis. We diagnosed 11(2.9%) TB cases, 8 (73%) of which were bacteriologically confirmed using Gene Xpert test. All the eleven clients began treatment and the community health workers followed them up. The elderly, (≥60 years) accounted for 37.5% of positive cases.

The effectiveness of a community wide active TB screening is widely debated and some of the reasons include; the high number of individuals that need to screen, the risk of having false-positive TB diagnoses and the unacceptable high number of patients who fail to start treatment [11]. We were able to deal with some of these concerns, though with some consideration.

The observed overall prevalence (2857/100,000) was about seven times as higher than the estimated prevalence of TB in the Ugandan general population, of 401/100,000[12]. The difference between our study findings and the Uganda national estimates may be explained by the fact that national figures include all age groups and all forms of TB. Secondly, the differences may to some extent be due to incomplete recording and reporting systems; the national notification data are often incomplete, leading to underestimation. Thirdly, the higher prevalence observed maybe an indicator of a high TB prevalence existing within Moroto, notably in the villages. In Moroto where the people live in “Manyattas” that are congested and poorly ventilated, ACF could be an applicable strategy.

The latest national prevalence survey in Uganda revealed a substantial burden of undetected TB, and about half of TB cases are missed every year considering that the TB program notifies about 41,000 TB cases per year [12]. We used a simple and sensitive symptom screening using the intensified case finding (ICF) form. This resulted in high numbers of presumptive cases and only 58.7% of the presumptive cases were able to produce sputum, which was analyzed using Gene Xpert. The presumptive cases, which turned out to be smear negative should ideally have been evaluated, further in the routine health system to rule out smear negative TB, given that it is fairly likely in high HIV settings such as Uganda. All the presumptive cases were advised to visit the hospital for a rigorous clinical evaluation as recommended under the new WHO practical Approach to Lung Health (PAL) strategy[13]. Although labor intensive, our approach was feasible because we relied on an existing well-functioning network of community health workers. Dakiko et al, has previously shown the important role of community health workers in improving access to TB diagnosis and care in active case finding in rural Ethiopia [14].

All our samples were analyzed using Gene Xpert, which has a higher sensitivity for *mycobacterium tuberculosis* (MTB) which enables it to detect the bacteria even in samples with a low bacillary load. However, sputum smear microscopy which is widely used in lower Health facilities to diagnose TB proves to have significant limitations since it can only detect TB in sputum with high bacillary load, thus leaves a significant number of cases undetected. Gene Xpert clearly outperforms smear microscopy in the few studies reporting on its use in ACF [12] [15,16]. Gene Xpert detected 72.7% (8/11) of the active TB cases in this study. Although in this study we used Gene Xpert for all the samples, its utility in active TB case finding in terms of performance, cost and impact requires further evaluation.

Active screening for TB ideally leads to improved case detection, shortened diagnostic delays and earlier treatment initiation[7]. First, referring to previous years, case notifications in the whole of Moroto increased especially with support from health-related Non-governmental Organizations.

The findings from this current study reflect the apparently low rate of detection, yet high rate of transmission and supports the complementary role of alternative case finding strategies such as ACF in similar communities in Uganda [12].

Secondly, we were able to give results to study participants with bacteriologically confirmed TB within a median of three days from sputum collection, which was a substantially shorter turnaround time than the generally reported 7–10 days delay especially for community experience. We relied mainly on the community health workers to follow up the positive clients and initiate them on treatment. In cases where the clients had telephone contacts, they were called to the facilities to start treatment. While we should have aimed at having same day issuing of results, the delay was due to the late arrival of the specimens in the laboratory and sometimes-high workload in the laboratory. Therefore, there is an urgent need for a real point of care test for TB. In addition, if infrastructure allows, further decentralization of TB diagnosis to the lower health facilities could contribute to reducing diagnostic delays.

Thirdly, the impact of ACF in improving case detection is best achieved by ensuring treatment initiation and completion. In contrast to reports from South Africa and India [15,17], treatment uptake in our study was high. This was due to the committed team of health workers who offer TB management care. However, patients delayed treatment for various reasons such as inability to find the patient, initial refusal of diagnosis, travel distance, need for hospitalization, inconvenient opening hours of health facilities or perceived cost of treatment.

Amidst so many challenges, household to household screening in our communities was operationally feasible and effective. Different interventions for community TB screening have been described including ACF through home visits or mobile clinics or enhanced case finding through home visits followed by referral to a health facility [18]. In the DETECTB-trial in Zimbabwe[11] the mobile van strategy outperformed door to door enquiry, whereas in t h e ZAMSTAR trial the household intervention proved more effective [19]. There might not be a single best strategy to fit all socio-cultural contexts for TB screening. The acceptability of home visits in our settings needs to be evaluated in more depth through qualitative research to inform implementation of plans.

## 5. CONCLUSION

Community based ACF through community health workers with targeted use of Gene Xpert proved feasible and effective in rural settlements of Moroto. ACF allowed these communities to timely access TB diagnostic services; it identified a substantial proportion of infectious cases and a flexible patient centered treatment approach ensured that most patients are initiated on treatment. Longer-term follow-up data are needed to evaluate the impact on TB transmission. Community wide TB screening could be considered as a complementary strategy to passive case detection in similar TB endemic settings. Given the achievements in several health focus areas, community health worker involvement is increasing, even in Uganda. For the successful contribution of the community health workers to be sustained, integration in the health system and formal recognition and remuneration must be pursued.

## 6. LIMITATIONS

Although our study had several strengths such as the implementation of community based ACF embedded in a well-functioning national TB programme and low initial lost to follow up rate, it also has limitations. Most of the household members were not available for interview, mostly the men who are involved in nomadic activities.

A good number of presumptive cases were unable to produce sputum and were referred to the facility for further assessment but many did not turn up probably due to long distances to the facility and lack of transport means. Those that turned out to be negative on Gene Xpert did not turn up for further work up. A more comprehensive diagnostic work-up of sputum negative clients is needed. There is need to get a mobile x-ray system to support in community diagnosis. A discussion on the sensitivity and specificity of Gene Xpert is beyond the scope of this study.

## Figures and Tables

**Fig. 1.**
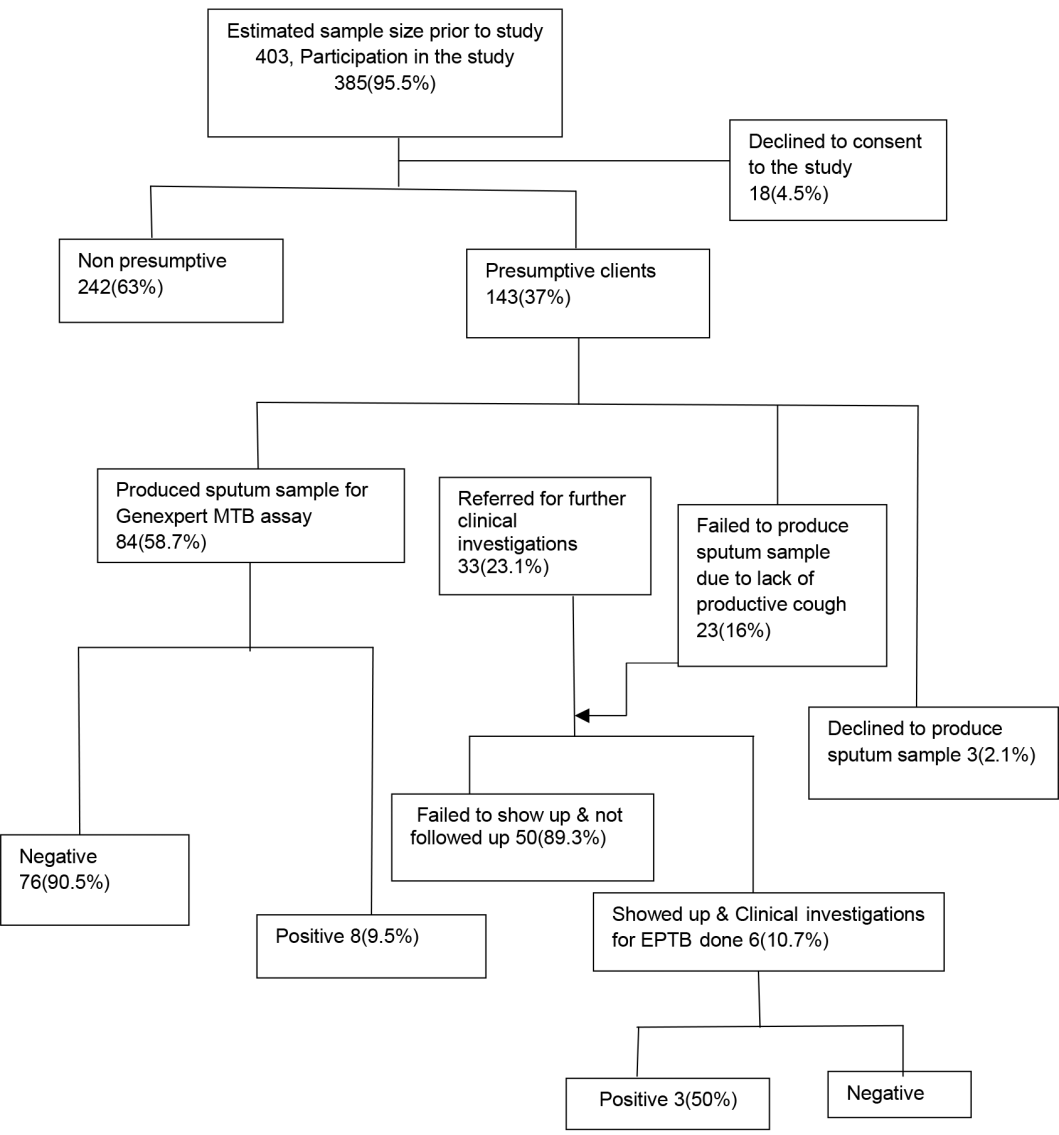
Study flow and tuberculosis yield

**Scheme. 1.**
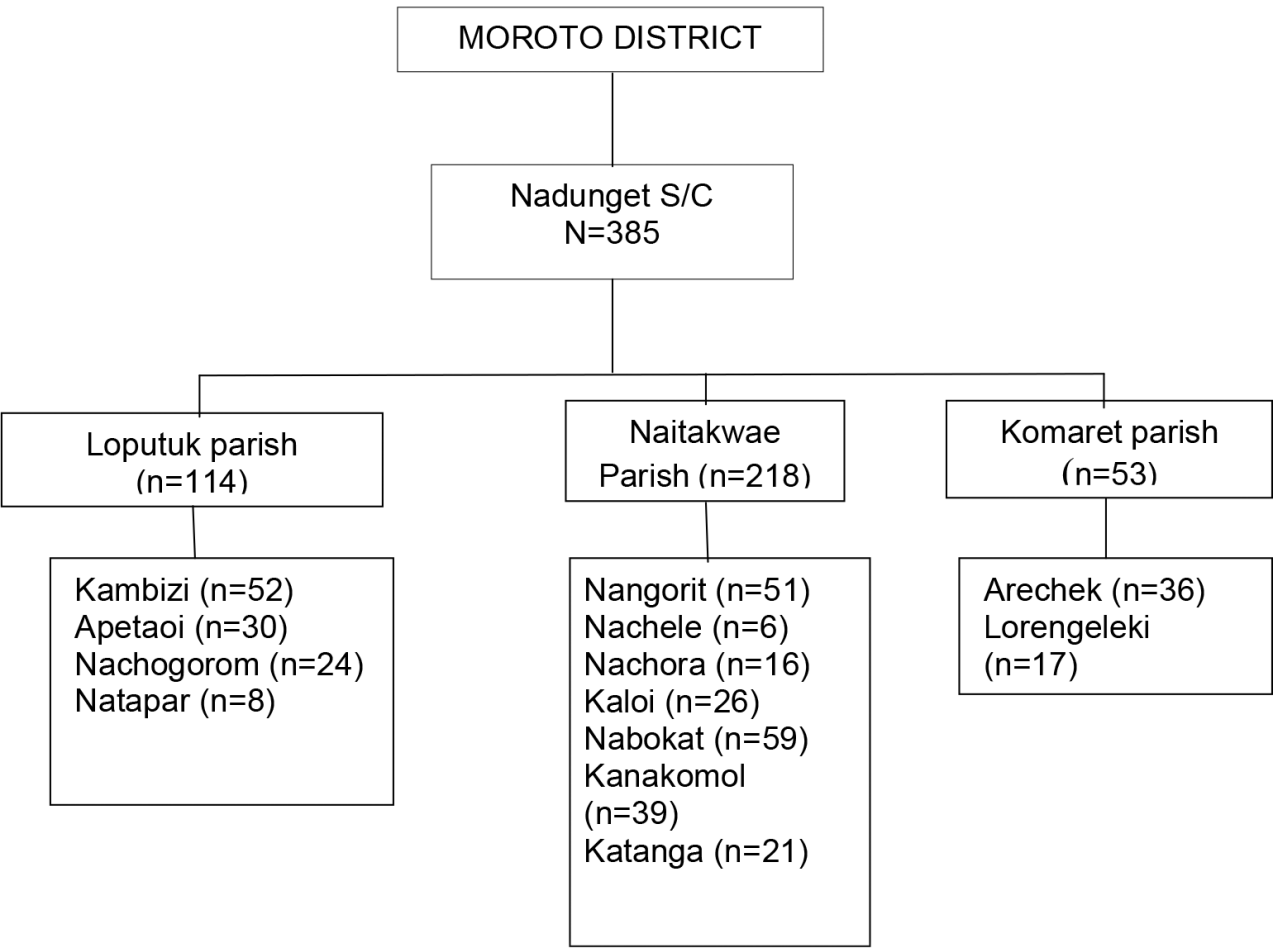
Sampling framework

**Table 1.** Demographic characteristics of the study population

Socio-demographic Characteristics	Frequency (%) N=385	Socio-demographic characteristic	Frequency (%) N=385
Gender		Religion	
Male	145(37.7)	Anglican	10(2.6)
Female	240(62.3)	Catholic	369(95.8)
Marital status		Moslem	3(0.8)
Cohabiting	3(0.8)	None	1(0.3)
Divorced	3(0.8)	Pentecostal	2(0.5)
Married	264(68.6)	Education level	
Single	62(16.1)	A ‘Level	2(0.5)
Widow	53(13.8)	Higher Institution	7(1.8)
Occupation		Never	264(68.6)
Peasant	62(16.1)	O’ Level	26(6.8)
Salary Earner	9(2.3)	Primary	86(22.3)
Self employed	32(8.3)	Housing Status	
Unemployed	258(67.0)	Permanent	8(2.0)
Wage earner	24(6.2)	Semi-permanent	88(22.9)
Religion		Temporary	289(75.1)
Anglican	10(2.6)	Family Type	
Catholic	369(95.8)	Extended	228(59.2)
Moslem	3(0.8)	Nuclear	157(40.8)
None	1(0.3)		
Pentecostal	2(0.5)		
